# Dermoid Cyst Spillage Resulting in Chemical Peritonitis: A Case Report and Literature Review

**DOI:** 10.7759/cureus.29151

**Published:** 2022-09-14

**Authors:** Adesola A Agboola, Khalid Uddin, Shafaq Taj, Greeshma Gopakumar, Chinyere L Anigbo, Hira Nasir, Muhammad Haseeb, Ayesha Javed

**Affiliations:** 1 Pathology and Laboratory Medicine, Dele Hospitals, Lagos, NGA; 2 Neurology, Henry Ford Health System, Detroit, USA; 3 Internal Medicine, Keystone Health/Penn State College of Medicine, Hershey, USA; 4 Internal Medicine, Larkin Community Hospital Palm Springs Campus, Hialeah, USA; 5 Internal Medicine, Father Muller Medical College, Mangalore, IND; 6 Internal Medicine, University of Nigeria, Enugu, NGA; 7 Internal Medicine, Mayo Hospital Lahore, Lahore, PAK; 8 Internal Medicine, Bahria International Hospital, Lahore, PAK

**Keywords:** chemical peritonitis, acute peritonitis, benign mature cystic teratoma, ovarian dermoid cyst, ovarian cyst

## Abstract

A dermoid cyst, also called a mature teratoma, is a benign tumor of the ovary derived from pluripotent germ cells. It is often asymptomatic; however, it can be expressed by several complications, including infection, adnexal torsion, and rupture. Rarely ovarian dermoid cysts can also transform into malignant degeneration. A ruptured teratoma is a rare and life-threatening complication and may arise spontaneously. However, cystic rupture is often secondary to surgical procedures such as ovarian cystectomy, leading to acute peritonitis and surgical emergency. Herein, we report a case of acute peritonitis in a female resulting from ovarian dermoid cyst spillage. Her clinical picture and radiological imaging were consistent with a ruptured ovarian cyst leading to chemical peritonitis, and a histopathological examination confirmed an ovarian dermoid cyst.

## Introduction

A mature teratoma belongs to a class of ovarian germ cells tumor. An ovarian dermoid cyst is a benign tumor of the ovary derived from embryonic stem cells. A dermoid cyst is a slow-growing tumor that grows from ectodermal components along the line of embryonic closure [1]. Mature teratoma is one of the most growing ovarian neoplasms, accounting for 15-20% of all neoplasms, with the highest prevalence in middle-aged women [2]. Ovarian dermoid cysts usually arise in middle-aged women and are asymptomatic. Manifestations include infection, adnexal torsion, rupture, and malignant degeneration depending on the size of the cyst, ranging from a few centimeters to 15cm [3]. Other complications include granulomatous nodules, dense adhesions, and hemorrhage leading to shock [3,4]. A ruptured ovarian dermoid leading to chemical peritonitis is a surgical emergency and a life-threatening complication. A dermoid cyst may rupture spontaneously or after surgical procedures such as ovarian adnexectomy [4]. Herein we report a rare case of chemical peritonitis resulting from ovarian ruptured teratoma spillage.

## Case presentation

**Figure 1:**
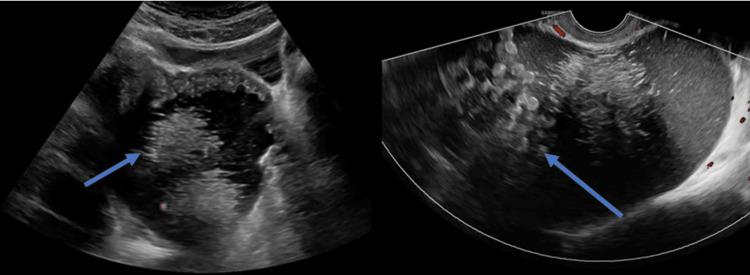
Pelvic ultrasonography showing right-sided ovarian cystic cavity with multiple small hyperechoic dots without internal flow (blue arrows).

A 51-year-old female was brought to the emergency department with a sudden onset of right lower quadrant pain for the last hour. The pain was progressive, sharp, non-radiating, and worsened on movement, with no relieving factors. Associated symptoms included nausea, fever, and one episode of vomiting containing liquid and bowel contents. She also reported an increase in abdominal girth over the past few months. The patient denied experiencing any genitourinary or gastrointestinal symptoms and had no history of trauma. On arrival, she was anxious, with a temperature of 100oF and a heart rate of 99 beats/minute. Her abdomen was enlarged with the examination positive for diffuse tenderness, guarding, rebound, and absent bowel sounds. Ultrasonographic examinations showed a large cystic mass in the pelvic cavity and an echogenic mass in the lower abdomen (Figure 1).

**Figure 2:**
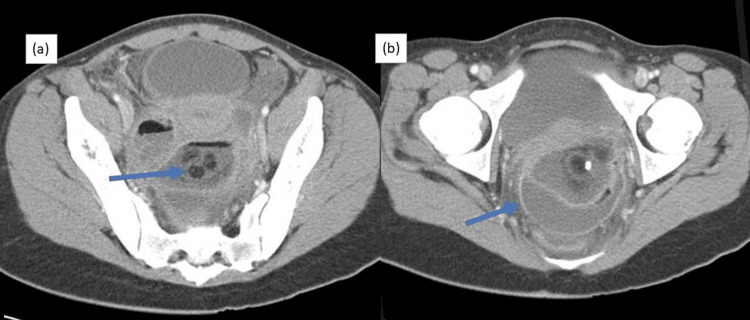
CT of the abdomen and pelvis showing the presence of three heterogenous rounded masses (a) and smooth enhancement of the peritoneal wall with free fluid in the abdominal cavity (b). CT, computed tomography

Computed tomography (CT) of the abdomen and pelvis was performed, which showed a large well-defined hypodense abdominopelvic cystic area measuring 6cm anteroposterior, 8cm transverse, and 4cm craniocaudal dimension with internal fluid level, and smooth enhancement of the peritoneal wall with free fluid in the abdominal cavity (Figure 2). Imaging was further enhanced to identify other pathologies such as adnexal torsion or ovarian neoplasm.

**Figure 3:**
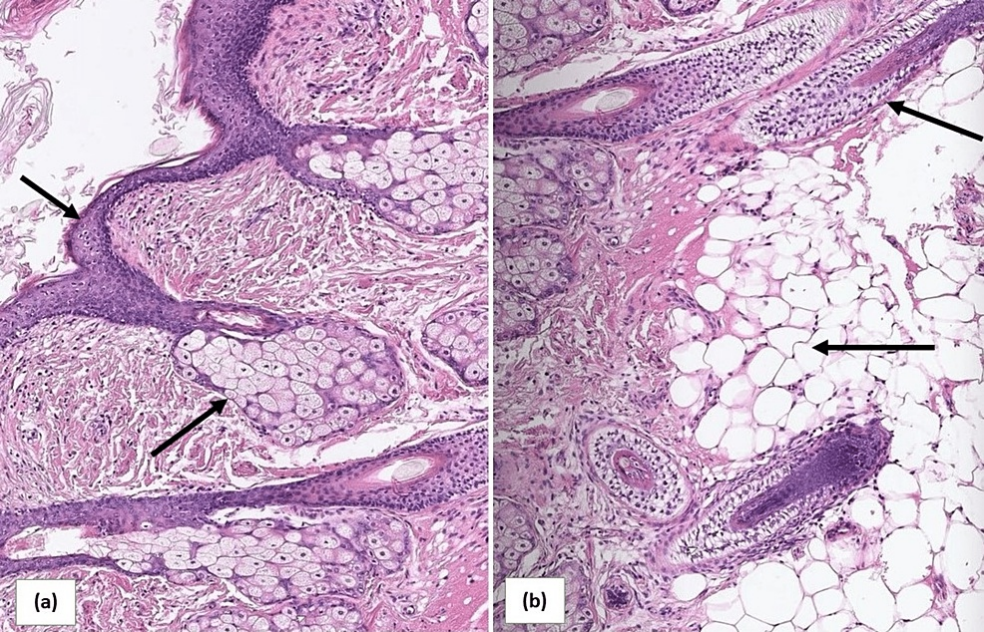
Histopathology demonstrating a complex cystic mass lined by epidermal cells containing hair follicles (a), fat cells, and glial cells (b).

The patient underwent surgical exploration due to concerns of peritonitis. On surgical exploration, cystic contents, including sebaceous material and hair fragments, were found along the septations. The ovaries were friable and bled easily, a portion of omentum was thickened and indurated, and cyst beds were loaded with dermoid cyst material. The removal of all dermoid cyst material was planned, with the excised right ovarian cyst sent for histopathological examination, revealing features consistent with ovarian teratoma (Figure 3). The postoperative period was uneventful, and the patient was started on broad-spectrum antibiotics and supportive management. Her condition improved, and she was discharged after four days.

## Discussion

Mature cystic teratoma is a subgroup of ovarian germ cell tumors. These benign tumors are further categorized into immature, mature, monodermal, and fetiform [1]. Mature cystic teratoma is the most common and comprises at least two or more well-differentiated germ cell layers [1,4]. It is termed a dermoid cyst if ectodermal components predominate the other germ cell layers. The origin of these tumors is usually in the midline and para-midline. In infants, these tumors arise in the sacrococcygeal region, while in adults, they predominantly arise in the gonadal region [5,6]. Malignancy from the dermoid cyst is rare and more common in children and old aged people. The thick wall of the dermoid cyst is made of keratinized squamous epithelium, which secretes sebaceous fluid, and sebum is the most characteristic finding of the dermoid cyst on imaging. Other cystic components include calcifications, bone, soft tissue, tooth, and hair [3,7].

Although rupture of ovarian cysts is not common, it may cause chemical peritonitis by leaking cystic contents into the peritoneal cavity. A minor dermoid cyst tear may lead to either abrupt or chronic rupture. In both cases, the patient may present with inflammatory nodules, ascites, tuberculous peritonitis, peritoneal carcinomatosis, or chemical peritonitis [8]. Chemical peritonitis can result in further complications, including fistula and adhesion formation. We have tabulated the reported cases of chemical peritonitis induced by ovarian dermoid cyst spillage (Table 1).

**Table 1:** Reported cases of peritonitis resulting from ovarian dermoid cyst spillage. USG, ultrasonography; CT, computed tomography

Study	Age	Clinical presentation	USG findings	CT findings	Surgical findings
Shamshirsaz et al. [7]	41	Anorexia, abdominal pain, fever	Bilateral cysts	Ascites, cystic mass	Adhesions, cystic mass, and cystic contents
Bužinskienė et al. [8]	35	Abdominal pain, vomiting, anorexia	Right multiloculated cyst, free fluid	Not reported	Ascites, cystic mass, and adhesions
Tsapralis et al. [6]	17	Diffuse abdominal pain	Hyperechoic right ovarian cystic mass	Not reported	Peritoneal adhesions, purulent fluid, ruptured cystic mass
Kim et al. [9]	35	Fever, abdominal pain	Not reported	Micronodules, ascites with peritoneal thickening	Adhesions, multiple nodules, ascites fluid, granulomas
Wong et al. [10]	35	Abdominal pain	Globular fatty locules	Bilateral ovarian cysts with fat fluid levels, calcifications, Rokitansky protuberance	Adhesions, sebum-like implants
Vulasala et al. [5]	33	Left lower quadrant pain	Large complex cystic and solid mass	Left ovarian mass with cystic and solid components, fat globules	Left ruptured cyst with free fluid in the peritoneal cavity
Li et al. [4]	66	Low-grade fever, abdominal pain	Complex mixed solid cystic mass	Right cystic mass containing fat, fluid, calcifications	Ruptured cyst with cystic contents, free fluid, and adhesions

Surgery is the definitive treatment of ovarian dermoid cysts. Even though laparoscopic procedure remains the conventional management for ovarian dermoid cysts compared to laparotomy due to its minimal invasiveness, the probability of causing peritonitis is increased in laparoscopy due to cyst contents spillage [11]. A study conducted in 2011 showed that cystectomy had a 60.32% risk rate during laparoscopic procedures, and adnexectomy had a 42.9% rupture rate. A direct correlation existed between ovarian cyst size and rupture risk, as 80% of ovarian dermoid cysts ruptured above the size of 60mm compared to the group with a diameter less than 60mm (51.17% of cysts ruptured) [12]. A dermoid cyst subgroup analysis highlighted a significant correlation between cyst rupture and chemical peritonitis (risk rate: 9.36; 95% confidence interval: 1.20-73.28) [13]. Introducing novel techniques, such as using a single port over a three port in laparoscopy, will significantly impact the rupture risk (3.0 vs. 22.2%); however, there will be no variations in postoperative complications [14]. The spillage of cystic contents can be prevented by covering the cyst first with a sterilized surgical sheet applied with quick-drying glue and then puncturing. This technique completely prevents the spillage of cyst fluid into the abdominal cavity [15].

## Conclusions

Pelvic pain in females has a comprehensive list of differentials. Diagnosing and managing a dermoid cyst in a deviant population comes with its own set of challenges. A ruptured teratoma is a rare and life-threatening complication. Although chemical peritonitis induced by ovarian dermoid cyst spillage is a rare complication, early recognition and prompt intervention by laparoscopy are essential in treating chemical peritonitis by removing the dermoid cyst and its contents.
